# Socioeconomic bias in influenza surveillance

**DOI:** 10.1371/journal.pcbi.1007941

**Published:** 2020-07-09

**Authors:** Samuel V. Scarpino, James G. Scott, Rosalind M. Eggo, Bruce Clements, Nedialko B. Dimitrov, Lauren Ancel Meyers

**Affiliations:** 1 Network Science Institute, Northeastern University, Boston, Massachusetts, United States of America; 2 Marine & Environmental Sciences, Northeastern University, Boston, Massachusetts, United States of America; 3 Physics, Northeastern University, Boston, Massachusetts, United States of America; 4 Health Sciences, Northeastern University, Boston, Massachusetts, United States of America; 5 ISI Foundation, Turin, Italy; 6 Department of Statistics and Data Sciences, The University of Texas at Austin, Austin, Texas, United States of America; 7 Department of Infectious Disease Epidemiology, London School of Hygiene and Tropical Medicine, London, United Kingdom; 8 Pediatric Healthcare Connection, Austin, Texas, United States of America; 9 Department of Operations Research, The University of Texas at Austin, Austin, Texas, United States of America; 10 Department of Integrative Biology, The University of Texas at Austin, Austin, Texas, United States of America; 11 Santa Fe Institute, Santa Fe, New Mexico, United States of America; The Pennsylvania State University, UNITED STATES

## Abstract

Individuals in low socioeconomic brackets are considered at-risk for developing influenza-related complications and often exhibit higher than average influenza-related hospitalization rates. This disparity has been attributed to various factors, including restricted access to preventative and therapeutic health care, limited sick leave, and household structure. Adequate influenza surveillance in these at-risk populations is a critical precursor to accurate risk assessments and effective intervention. However, the United States of America’s primary national influenza surveillance system (ILINet) monitors outpatient healthcare providers, which may be largely inaccessible to lower socioeconomic populations. Recent initiatives to incorporate Internet-source and hospital electronic medical records data into surveillance systems seek to improve the timeliness, coverage, and accuracy of outbreak detection and situational awareness. Here, we use a flexible statistical framework for integrating multiple surveillance data sources to evaluate the adequacy of traditional (ILINet) and next generation (BioSense 2.0 and Google Flu Trends) data for situational awareness of influenza across poverty levels. We find that ZIP Codes in the highest poverty quartile are a critical vulnerability for ILINet that the integration of next generation data fails to ameliorate.

## Author summary

Public health agencies maintain increasingly sophisticated surveillance systems, which integrate diverse data streams within limited budgets. Here we develop a method to design robust and efficient forecasting systems for influenza hospitalizations. With these forecasting models, we find support for a key data gap namely that the USA’s public health surveillance data sets are much more representative of higher socioeconomic sub-populations and perform poorly for the most at-risk communities. Thus, our study highlights another related socioeconomic inequity—a reduced capability to monitor outbreaks in at-risk populations—which impedes effective public health interventions.

## Introduction

As part of a broader national security strategy, US President Obama created the first *National Strategy for Biosurveillance*, outlining the nation’s key strategic goals in disease surveillance [1]. As a core component of this strategy, President Obama listed taking “full advantage of the advanced technologies… that can keep our citizens safe.” The surveillance systems outlined by the president are targeted at both recurring diseases, such as influenza, and newly emerging infections. Biosurveillance using advanced technologies may be most important in lower socioeconomic areas, where influenza burden tends to be highest [2–4].

This article assesses the capacity for traditional and novel data sources to provide real-time influenza risk assessments in under-served populations. Using a combination of public health, health care, and Internet-source data available between 2007 and 2012 to make short-term predictions of influenza-related hospitalizations, we compare forecasting accuracy across socioeconomic groups in the Dallas-Fort Worth metro area of Texas, USA. Traditional influenza surveillance is based on primary healthcare provider reports, which may be biased towards serving populations with higher socioeconomic status because of the costs and accessibility of healthcare [5, 6]. Next generation data sources provide promise for improving the timeliness and statistical power of surveillance systems. However, a systematic evaluation of the current surveillance system is needed to evaluate where it falls short, and whether new data can fill gaps.

New technologies have fueled a rapid expansion of data sources that can be acquired quickly and inexpensively for public health surveillance. For example, Google Flu Trends used Internet search queries of influenza-related terms for surveillance [7]. Following the introduction of Google Flu Trends, digital disease surveillance has exploded [8–10] with efforts focused on data from search engines [11, 12], crowd-sourced participatory surveillance (e.g., Flu Near You, InfluenzaNet) [13–15], Twitter (e.g., MappyHealth) [16, 17], Facebook [18, 19], Wikipedia access logs [20, 21], and a variety of other sources (as reviewed in [22, 23]). There is evidence that essentially all of these next-generation surveillance data streams correlate to some degree with epidemiological time-series during typical seasonal outbreaks.

However, there are at least two recent findings worth considering with respect to the these high-tech surveillance systems: 1.) the performance of Google Flu Trends has been unreliable during anomalous influenza outbreaks [24–26] and 2:) it is unclear who is responsible for maintaining these systems [22], especially considering that Google Flu Trends was recently taken offline.

Newly upgraded hospital information systems are another promising source of surveillance data. For example, the United States Centers for Disease Control and Prevention (CDC) launched the BioSense 2.0 program, a set of cooperative agreements between the Department of Veterans Affairs, the Department of Defense, and civilian hospitals from around the country. Through the cooperative agreements, the BioSense 2.0 program creates a “collaborative data exchange system that allows users to track health issues as they evolve” [27]. Whereas BioSense 2.0 provides real-time data on severe cases, the CDC’s primary influenza surveillance system, the influenza-like-illness network (ILINet), provides weekly estimates of number of patients presenting with influenza-like-illness symptoms at primary care clinics. Integrating potentially complementary information from new and traditional systems like BioSense 2.0 and ILINet, along with publicly available Internet-source data, like Google Flu Trends, may provide a more timely, comprehensive, and robust picture of disease activity. To this end, the Defense Threat Reduction Agency has begun a national effort to build the Biosurveillance Ecosystem, an integrated disease surveillance system providing access to diverse data sources and powerful analytics [28].

Here, we build and evaluate a multi-source influenza surveillance system that leverages traditional surveillance, electronic health records, and Internet-source data. It is designed to provide short-term forecasts of influenza-related inpatient hospitalizations once an epidemic is underway rather that provide early warning of emerging influenza threats. At the state and multi-county regional levels, these data sources provide effective situational awareness (as compared to early detection of outbreaks). However, we find that they are much more representative of higher socioeconomic sub-populations and perform poorly for the most at-risk communities. Thus, the integration of Internet and electronic medical records data into surveillance systems may improve timeliness and accuracy, but fail to remedy a critical surveillance bias.

## Materials and methods

### Ethics statement

The Texas Department of State Health Services Institutional Review Board #1 approved this project. The associated reference number is IRB# 12-051. An informed consent waiver was approved by the IRB.

### Data sources

We used the following sources, which contained data primarily from Dallas, Tarrant, Denton, Ellis, Johnson, and Parker counties in Texas, between 2007 and 2012:

1. Weekly BioSense 2.0 data were extracted from an online repository [29]. Data are the percent of emergency department (ED) visits for upper respiratory infection, based on classification of free-text chief complaint entries. Although ZIP Code level data are available, we used county-level aggregates in our analysis. Because these data are hosted on a publicly accessible site, we make them available in a CSV file hosted here: https://github.com/Emergent-Epidemics/US_influenza_data_1998_09-2019.
2. ILINet gathers data from thousands of healthcare providers across the USA. Throughout influenza season, participating providers are asked to report weekly the number of cases of influenza-like illness treated and total number of patients seen, by age group. The case definition requires fever in excess of 100°F with a cough and/or a sore throat without another known cause. The Texas Department of State Health Services (DSHS) provided weekly ILINet records from 2007–2012. In the main text, we use county-level aggregates and provide results with ZIP Code level aggregates in S5 Text.
3. Google Flu Trends (GFT) estimated the number of ILI patients per 100, 000 people based on the daily number of Google search terms associated with signs, symptoms, and treatment for acute respiratory infections. Although GFT is no longer active, past data are available for download from Google.org and have been shown to reliably estimate seasonal influenza activity [7, 30], but be unreliable for the 2009 H1N1 pandemic [31] and during more recent influenza seasons [25]. We considered six different GFT time series, corresponding to the state of Texas and five cities in the Dallas-Fort Worth area: Fort Worth (Tarrant county), Irving (Dallas county), Plano (Collin and Denton counties), Addison (Dallas county) and Dallas (Dallas county). Google searches are geo-located using the IP address of the device [7]. We used one state-level and six city-level GFT data in all models.

The surveillance models predict hospitalizations that have been aggregated by income quartile. We obtained hospital discharge records from Texas Health Care Information Collection (THCIC), filtered for influenza-related principal diagnostic codes of ICD-9 487.*, which includes 487.0 (with pneumonia), 487.1 (with other respiratory manifestations) and 487.8 (with other manifestations). The data are aggregated into weeks and by patient ZIP Code. Patient ZIP Codes were then combined into income quartiles based on US Census estimates.

**Fig 1:**
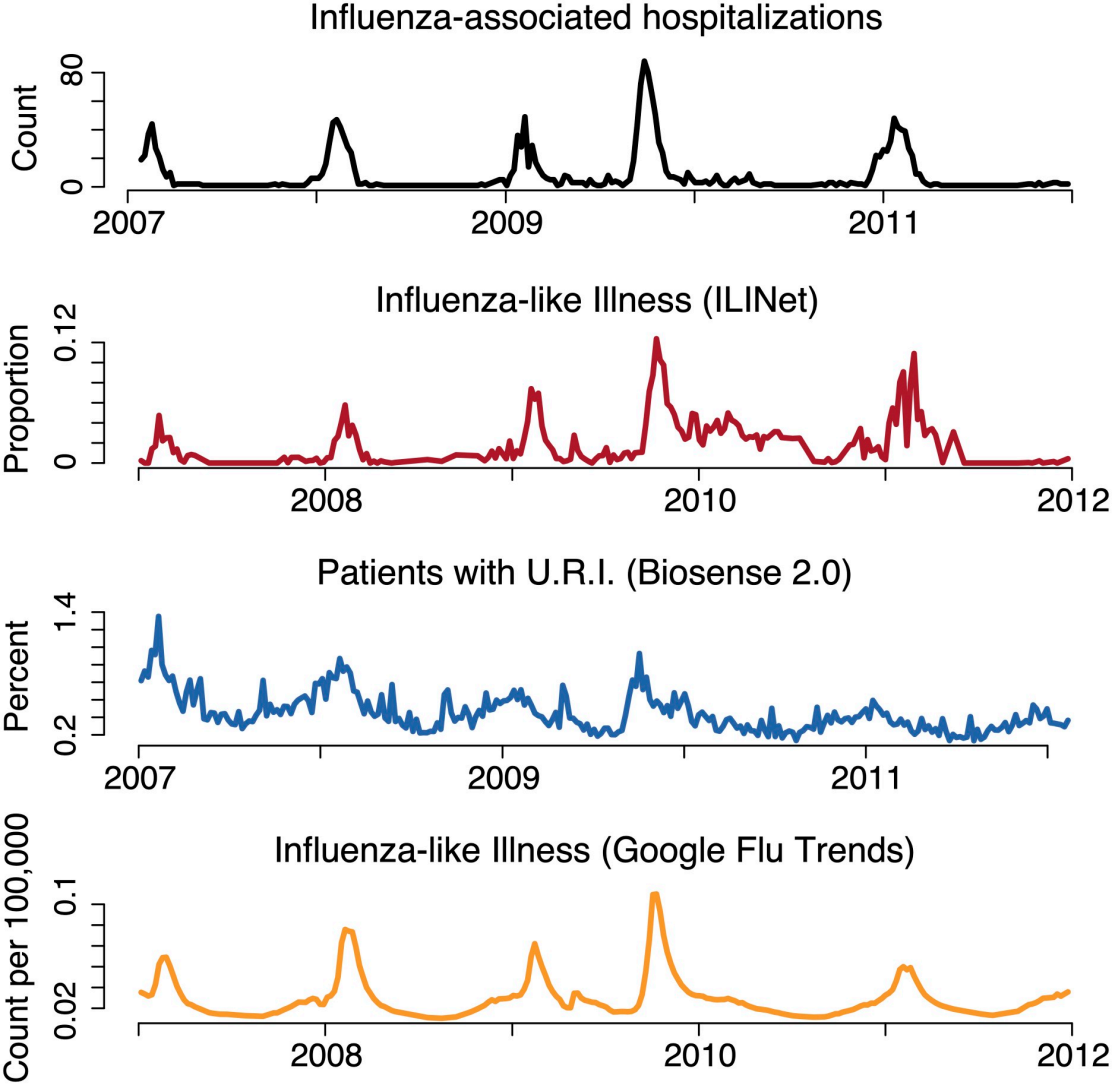
Datasets used in the analysis. The top panel shows influenza-associated inpatient hospitalizations in black, as defined by ICD9 codes 486 and 487, the next panel shows ILINet in red where the proportion of doctor visits are for influenza-like illness, the next panel shows BioSense 2.0 in blue, which is the proportion of ED visits per week that are for an upper respiratory infection. The final panel shows the GFT estimate, in orange, of the number of influenza-like-illness cases per 100,000 people.

Fig 1 presents aggregate counts from the BioSense 2.0, ILINet, GFT, and hospitalization data used in the study for the Dallas-Fort Worth region. We grouped ZIP Codes into quartiles, based on the percentage of the population living in poverty reported in the 2011 American Community Survey [32]. We estimated age distributions within ZIP Codes from the 2011 American Community Survey and the 2010 Census.

### Short term predictions

We used generalized additive models to make short-term predictions of influenza-related hospitalizations in the study populations. First, we partitioned ZIP Codes into four poverty quartiles. To predict hospitalizations for group *i*, we use the Poisson generalized linear model given by

$$\begin{array}{c} y_{t}^{\left(i\right)}∼\text{Poisson}\left(\lambda_{t}^{\left(i\right)}\right)\;,\;\log\lambda_{t}^{\left(i\right)}=\alpha^{\left(i\right)}+\sum_{k=1}^{D}h_{k}^{\left(i\right)}\left(x_{k,t}\right)\;, \end{array}$$ (1)

where $y_{t}^{\left(i\right)}$ is the total number of hospitalizations in group *i* at time *t*, *x*_*k*,*t*_ is the *k*th predictor for hospitalizations at time *t*, *α*_*i*_ is a background hospitalization rate for group *i*, and $h_{k}^{\left(i\right)}\left(\cdot \right)$ is some potentially nonlinear function (specific to group *i*) that maps predictors to expected hospitalization counts. Intuitively, the *x*_*k*,*t*_ scalars capture all the information used by the surveillance model to predict hospitalizations. Here *t* indexes the time of the prediction and *k* the particular data source—for example, GFT data from two weeks prior. We fit the $h_{k}^{\left(i\right)}\left(\cdot \right)$ by expanding each predictor in a third-order B-spline basis with six degrees of freedom. The result of this expansion is that each predictor is now also represented by a number of new predictors, which functionally allow for non-linear associations between the original predictors and influenza hospitalizations. To avoid overfitting, we regularized the spline coefficients using a lasso penalty, with the regularization parameter chosen by cross-validation.

Let *y*_*i*_ = (*y*_*i*1_, …, *y*_*iN*_)^*T*^ be a vector of counts for income quartile *i* across all weeks. Let *X* be an *N* × *D* matrix of surveillance variables used as predictors, where rows are weeks and columns are variables. We considered one-week-ahead forecasts, thus entry *t* in *y*_*i*_ corresponds to this week’s hospitalization count, while row *t* of the *X* matrix (used to forecast *y*_*it*_) corresponds to information based on surveillance variables up through week *t* − 1 only. Two-week-ahead forecasts were similar, but with the *X* matrix containing data only through week *t* − 2.

We considered six different model variations, each using a distinct combination of data from BioSense 2.0, ILINet, and GFT. Importantly, these three data sets included multiple time series. For example, BioSense 2.0 provided hospitalization counts for all six counties in the study area. Additionally, for each time series we added three columns in the *X* matrix: the level (actual value of the time series in the trailing week), the slope of that variable (first difference over the trailing two weeks at the time of prediction), and the acceleration (second difference over the trailing three weeks at the time of prediction). The columns of *X* corresponded to the predictors in the model, and we considered six sets of predictors: (i) ILINet alone (15 predictors), (ii) BioSense 2.0 alone (18 predictors), (iii) GFT alone (18 predictors), (iii) ILINet+ BioSense 2.0 (33 predictors), (iv) ILI + GFT (33 predictors), (v) BioSense + GFT (36 predictors) and (vi) GFT + ILINet+ BioSense 2.0 (51 predictors). In addition, the B-spline expansions provided another 6 variables for each set of predictors, for example, the fully expanded version of model (vi) would have 51 + 306 predictors for a total of 357.

Across the 6 models, we fitted separate models to each group *i*; these group-level models shared the same predictors, but result in different regression coefficients from B-spline expansions of each partial response function. Overall, we fitted 16 models, one for every combination of ZIP Code group (*i*) and candidate predictor set described. Given that we had 188 weeks of data and between 15 and 357 predictors per model, we regularized the coefficient estimates in order to avoid over-fitting. Specifically, we applied a lasso penalty on the coefficient vector *β*, by minimizing the objective function

$$\begin{array}{c} f(\beta )=l(\beta )+\lambda \;p(\beta )\;, \end{array}$$

where *l*(*β*) is the negative log likelihood arising from the Poisson model, *p*(*β*) is the lasso penalty function, and λ is a scalar that governs the strength of regularization. We select λ for each regression separately using cross validation. See [33] for further details of the model-fitting algorithm. A similar procedure to avoid over-fitting associated with influenza forecasting was utilized by [34].

### Predictive performance

To evaluate the predictive performance of the models, we calculated out-of-sample RMSE (ORMSE). Let ${\hat{y}}_{it}$ be the predicted hospitalization count for group *i* on week *t*, generated from fitting the model to every data point except week *t*. The quantity

$$\begin{array}{c} e_{it}=y_{it}-{\hat{y}}_{it} \end{array}$$

is the out-of-sample prediction error. We refitted the model 188 times, one for each week that is removed; this is repeated for every group and every combination of surveillance variables. The ORMSE for a group of ZIP Codes *i* is given by

$$\begin{array}{c} {\text{ORMSE}}^{\left(i\right)}=\frac{\sqrt{\frac{1}{N}\sum_{t=1}^{N}e_{it}^{2}}}{{\text{Pop}}^{\left(i\right)}}\;, \end{array}$$

where *N* is the number of weeks, and Pop^(*i*)^ is the total population of the group. This can be interpreted as the average predictive error of the model. The units are hospitalization counts per person. Although the groups have approximately the same population size, normalizing by the population of the group is essential. Without normalization, predictions for a large population may appear worse than predictions for a small population, simply because more hospitalizations occur in the larger group. We corroborated our ORMSE results using a log-likelihood analysis (see S2 Text).

To determine whether performance differences between poverty groups were statistically significant, we ran a permutation test with 10,000 repeats, by randomly assigning ZIP Codes into four equally sized groups, and re-fitting the model to each randomized group, following the original procedure, including cross-validation regularization. We then calculated ORMSE for each group, and also the difference between the best ORMSE and the worst ORMSE among the four groups.

For each of the four model variants, we (1) used this procedure to generate null distributions of test statistics for each of our four model variants, (2) calculated the difference between the ORMSE measured for the highest poverty quartile and that measure in the lowest poverty quartiles (according to the original grouping), and (3) determined the proportion of the null distribution less than this difference. This proportion was the *P*-value used to determine statistical significance.

## Results

**Fig 2:**
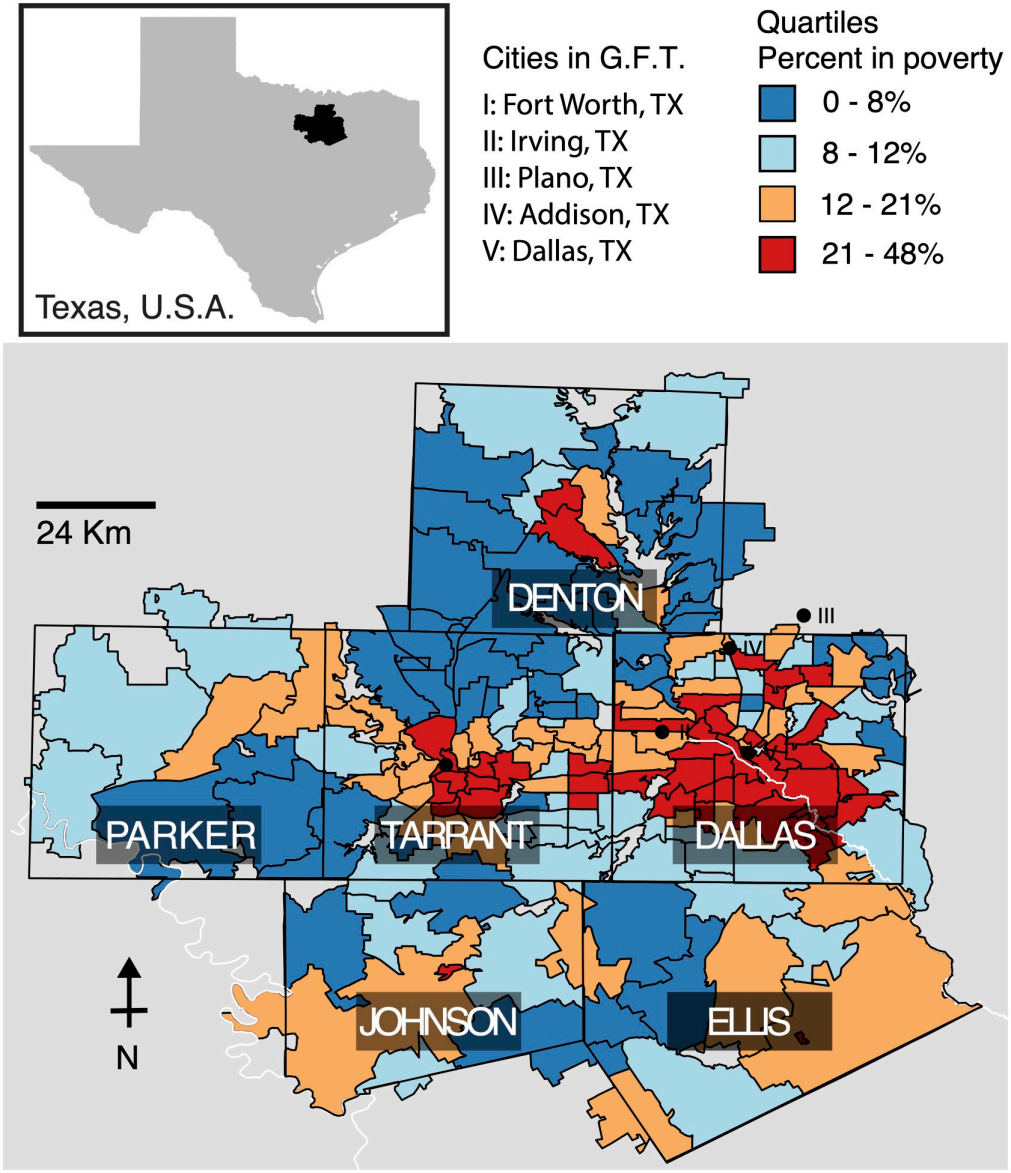
The six counties in northeast Texas included in this study (Dallas, Tarrant, Parker, Denton, Johnson, and Ellis). Zip codes are colored by their poverty quartile, [0-8) (dark blue), [8-12) (light blue), [12-21) (orange), >21 (red) percent of residents below the poverty line. In addition to the state-level Google Flu Trends (GFT) time series, we used the five city-level time series most closely associated with our study area: Fort Worth (I), Irving (II), Plano (III), Addision (IV), and Dallas (V).

We evaluated the performance of BioSense 2.0, GFT and ILINet data sources, with respect to short-term predictions of influenza-related hospitalizations in the six-county region surrounding the Dallas-Fort Worth metropolitan area (Fig 2). This region included 305 ZIP Codes and all of the emergency departments reporting to the Texas BioSense 2.0 system during the five-year study period (2007-2012).

### Influenza burden by poverty level and age

**Fig 3:**
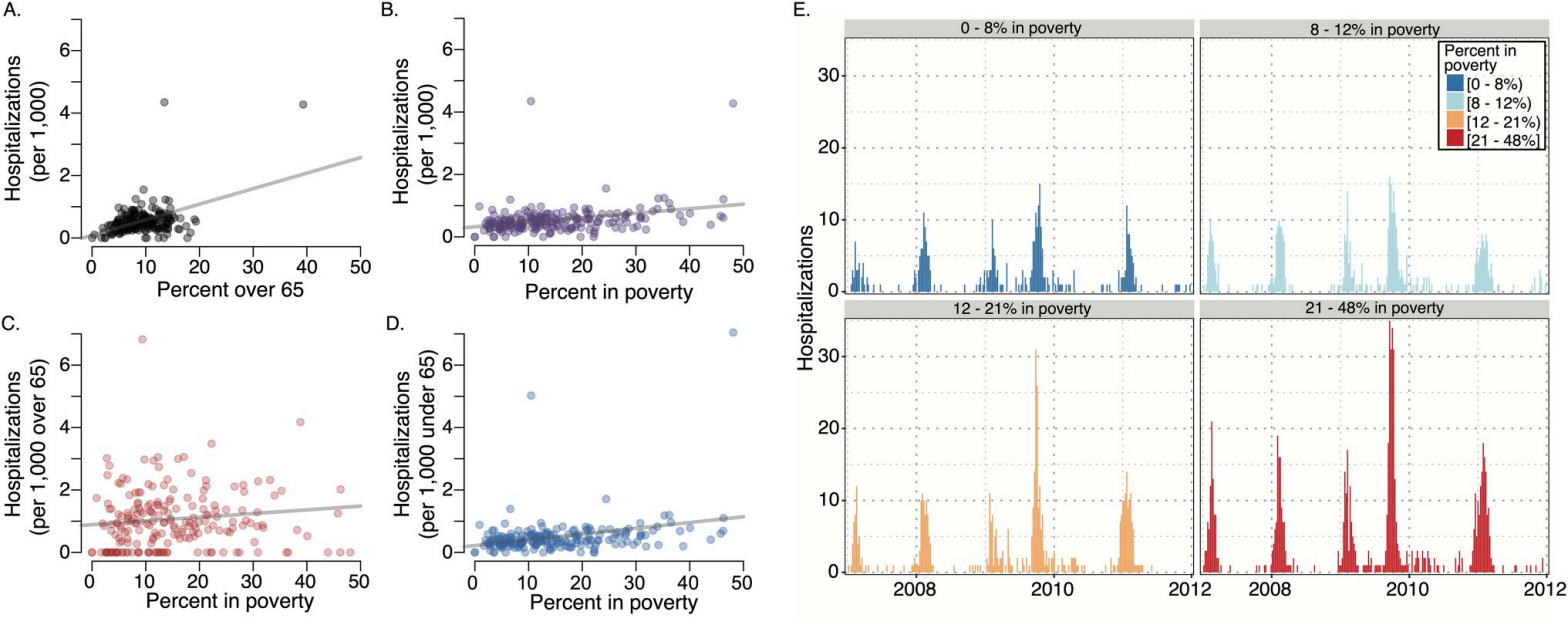
Relationship between age, poverty level, and influenza hospitalizations across 305 ZIP Codes from 2007 to 2012. Demographic data are based on 2010 Census. (A) Influenza hospitalizations increase with the size of the over 65 population (p <.001). (B) Influenza hospitalizations increase with the percent of the population under the federal poverty level (p <.001). (C) Influenza hospitalizations in over 65 year olds does not significantly increase with poverty (p = .11). (D) Influenza hospitalizations in under 65 year olds does significantly increase with poverty (p <.001). (E) The weekly number of hospitalizations across each of the four poverty quartiles. Because the quartiles were selected to include comparable population sizes, we find 2—3 times higher rates of inpatient hospitalizations in the most impoverished quartile (red) as compared to the least (dark blue).

We estimated the influenza hospitalization rate per 1,000 people in each ZIP Code. Throughout the region, we find that influenza hospitalization rates exhibit a significant positive correlation with both poverty level and the proportion of the 2010 census population over age 65 (Fig 3), consistent with recent literature [2, 3, 35]. After controlling for age, we find that poverty and influenza burden are significantly correlated in the under age 65 population but not the over age 65 population (2011 American Community Survey estimates) (*p*<.001). We established this result with a multivariate regression of hospitalization rate at the zip-code level with the proportion of the ZIP Code living below the poverty line and the proportion of the ZIP Code over 65 (Table 1).

**Table 1:** A multivariate regression of hospitalization rate at the zip-code level with the proportion of the ZIP Code living below the poverty line and the proportion of the ZIP Code over 65.

	Estimate	Std. Error	t value	Pr(>|t|)
(Intercept)	0.3656	0.0978	3.74	0.0002
Proportion over 65	-0.0001	0.0094	-0.01	0.9911
Proportion in poverty	-0.0096	0.0043	-2.25	0.0258
Interaction	0.0022	0.0003	6.26	0.0000

### Forecast quality by poverty quartile

**Fig 4:**
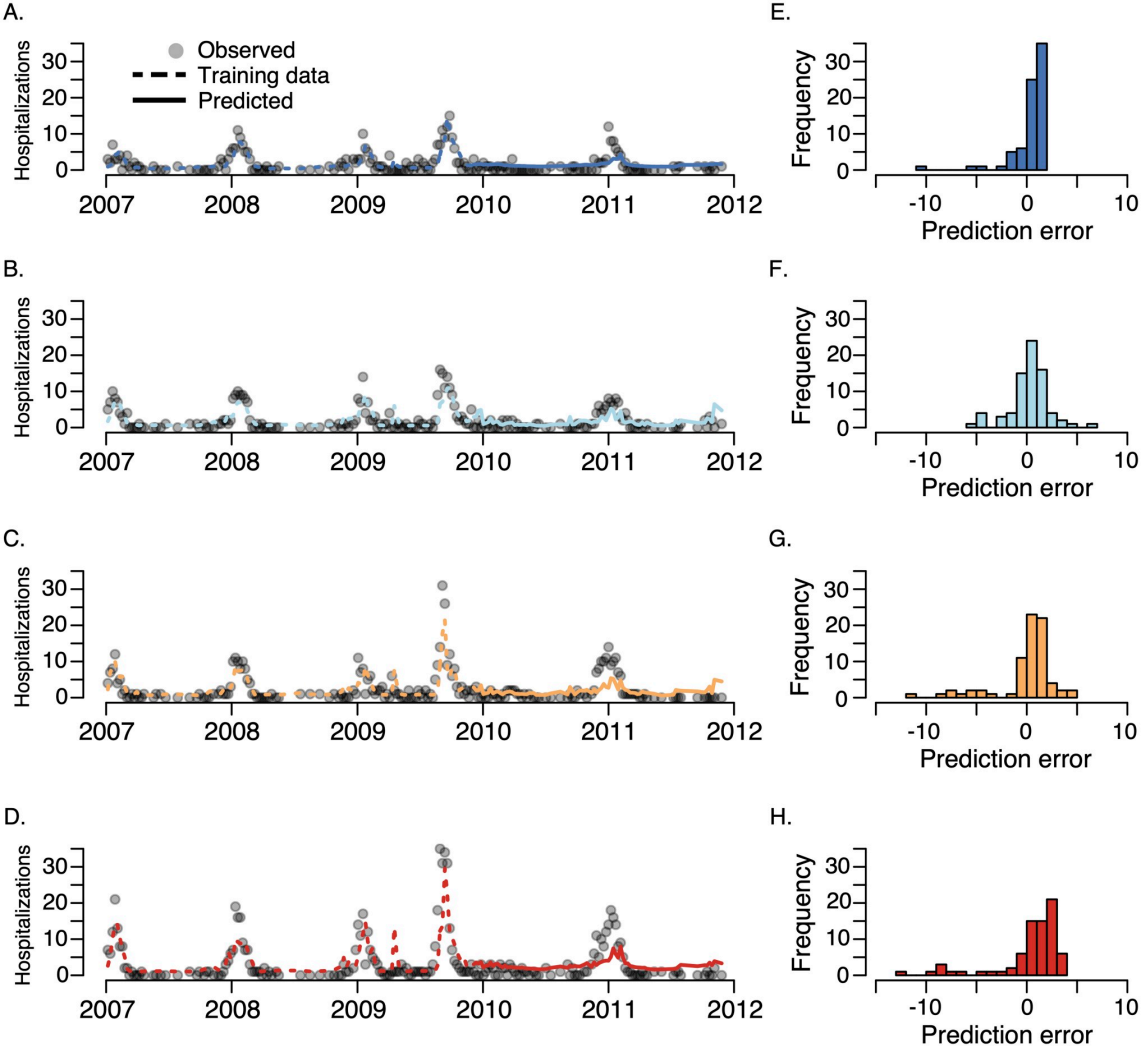
Comparison between one-week ahead model predictions and the total number of weekly observed influenza hospitalizations for each of the four poverty quartiles (A) upper quartile (i.e. least impoverished), (B) upper-middle quartile, (C) lower-middle quartile, (D) lowest quartile (most impoverished) and the distribution of out-of-sample prediction errors (observed—predicted) for the (E) upper quartile, (F) upper-middle quartile, (G) lower-middle quartile, and (H) lowest quartile. The model was trained on the first 60% of the data (dashed lines) and evaluated on the remaining 40% of the data (solid lines). Qualitatively similar results were obtained with n-fold (leave-one-out) cross-validation, see Tables 2 and 3 and S2 Text. Across all four quartiles, the model was unbiased according to a re-sampling test on the residuals, see S3 Text.

We classified ZIP Codes into quartiles based on the proportion of the population living below the federally defined poverty line and fitted separate generalized additive forecasting models to the data in each of the quartiles. In comparisons between model predictions and hospitalization data, we find that the data become less informative as the poverty level increases (Fig 4 and Tables 2 and 3). The models make the best predictions in the most affluent 25% of ZIP Codes—with poverty levels between 0% and 7.5%— and the worst predictions in the most impoverished 25% of ZIP Codes—those with poverty levels between 21.2% and 48.1%, regardless of the data sources included as predictors. Additionally, in an attempt to reduce the forecasting bias, we included the out-of-sample predictions for the three lower poverty quartiles as candidate predictors for highest poverty quartile. This model did not improve the forecast accuracy in the highest poverty quartile (see S1 Text). The differences in prediction errors between the upper and lower poverty quartiles are statistically significant (*P* < 0.0001, bootstrap analysis and S1 Fig). This trend is confirmed by generalized linear Poisson and negative binomial models–along with generalized additive Poisson and Gaussian models–fit using one-week-ahead forecasts and evaluated using leave-one-out root-mean-square-error and log-likelihood (all evaluated on out-of-sample data, see S2 Text and S4 Fig). Finally, to address potential biases arising from aggregating of ILINet data from ZIP code level to county level, we re-ran the analyses using ZIP Code level time ILINet time series. We again found that out-of-sample forecast accuracy was lowest in the most impoverished 25% of ZIP Codes, (see S5 Text).

**Table 2:** Out-of-sample (60/40 training/testing) root mean-squared error (ORMSE) using a Poisson generalized additive model. Values are normalized by the population size of each ZIP Code quartile and then multiplied by 10^6^ to obtain ORMSE per one million residents. The rightmost column gives aggregate ORMSE across all ZIP Codes included in our study area. The quartiles contained: [0-8) (1st quartile), [8-12) (2nd quartile), [12-21) (3rd quartile), and >21 (4th quartile) percent of residents below the poverty line.

Surveillance Data Sources	1st quartile	2nd quartile	3rd quartile	4th quartile	Combined
ILI	1.69	2.41	2.29	5.12	2.22
BioSense	1.55	1.95	2.51	2.60	2.01
GFT	1.38	1.34	2.16	2.68	1.74
ILI + BioSense	1.46	1.68	2.30	3.81	1.94
ILI + GFT	1.42	1.35	2.17	2.75	1.74
BioSense + GFT	1.44	1.58	2.11	2.64	1.79
ILI + BioSense + GFT	1.44	1.53	2.12	2.64	1.72

**Table 3:** Out-of-sample (leave-one-out) root mean-squared error (ORMSE) for each Poisson generalized additive model. Values are normalized by the population size of each ZIP Code quartile and then multiplied by 10^6^ to obtain ORMSE per one million residents. The rightmost column gives aggregate ORMSE across all ZIP Codes included in our study area. The quartiles contained: [0-8) (1st quartile), [8-12) (2nd quartile), [12-21) (3rd quartile), and >21 (4th quartile) percent of residents below the poverty line.

Surveillance Data Sources	1st quartile	2nd quartile	3rd quartile	4th quartile	Combined
ILINet	1.45	1.81	2.63	4.04	2.20
BioSense 2.0	1.69	2.05	2.66	4.23	2.52
GFT	1.33	1.64	2.61	3.63	2.00
ILINet+ BioSense 2.0	1.55	1.81	2.49	3.88	2.18
ILINet+ GFT	1.33	1.59	2.65	3.29	2.00
BioSense 2.0 + GFT	1.39	1.91	2.59	4.03	2.35
ILINet+ BioSense 2.0 + GFT	1.39	1.91	2.52	4.03	2.35

### Synchrony within poverty quartiles

One possible explanation for the observed bias in forecast accuracy by income quartile is that the most impoverished ZIP Codes are either out-of-sync with each other or are more widely distributed across the study area. We tested the hypothesis that the most disadvantaged quartile exhibits greater asynchrony in influenza hospitalization rates and/or are located further away from each other and found the opposite: ZIP Codes in the most impoverished quartile are more synchronous and are located no less closely together as compared to more affluent ZIP Codes.

**Fig 5:**
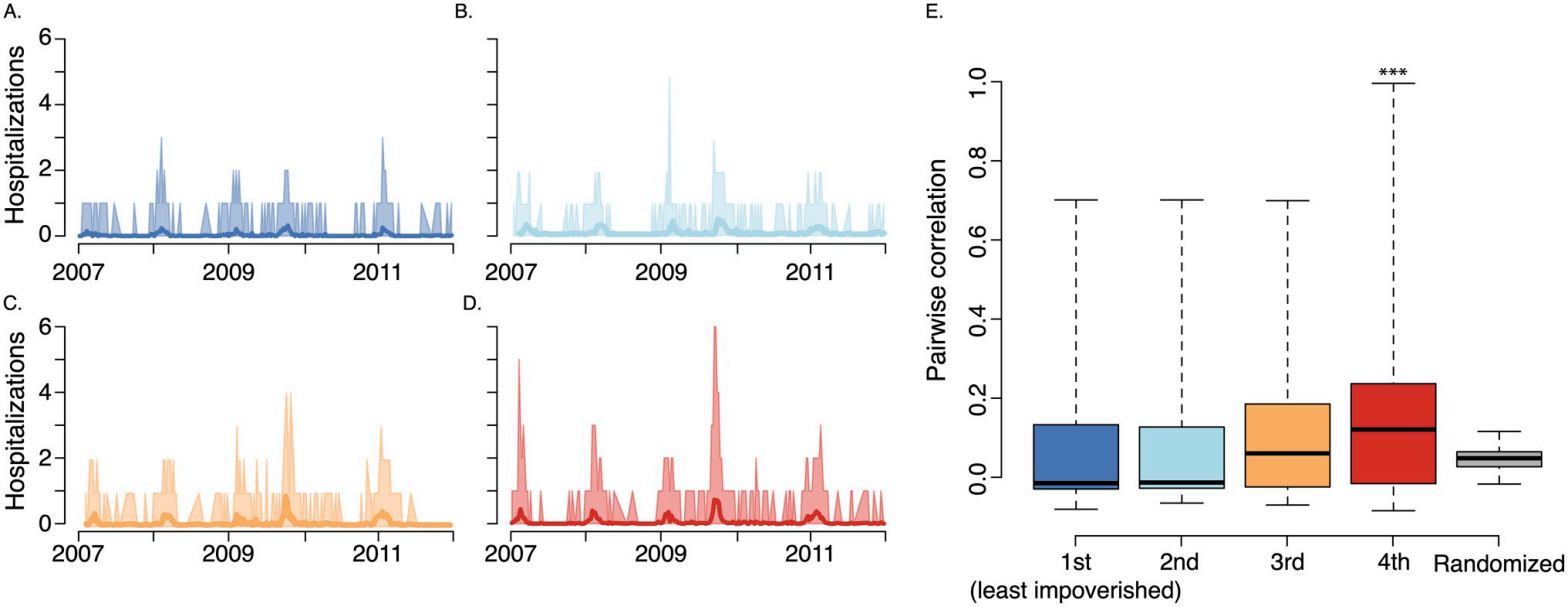
Influenza synchrony among ZIP Codes within each poverty quantile. The quartiles contained: [0-8) (A., 1st quartile), [8-12) (B., 2nd quartile), [12-21) (C., 3rd quartile), and >21 (D., 4th quartile) percent of residents below the poverty line. (A-D) Range of influenza activity (shades) around mean (solid line) at the ZIP Code level, in least impoverished to most impoverished quartiles, respectively. (E) Boxplots of correlation coefficient among pairs of ZIP Codes within each quartile. The most impoverished quartile exhibited the greatest synchrony. To test for significance, we randomly assigned ZIP Codes to income quartiles 5,000 times and repeated the analysis. The observed mean correlation among the most impoverished quartiles was higher than all of the 5,000 randomizations (i.e., *p* < 0.0002) and the observed median was higher than all but 2 of the simulations (i.e., *p* = 0.0004). The median value for each quartile is indicated with a solid, black line, the boxes enclosed the inter-quartile range, and the whiskers cover the entier distribution. We include the distribution of randomized median correlations on the far right (gray box plot). As discussed in the Results, we confirmed these results using a principle component analysis.

We define asynchrony as the average pair-wise correlation between ZIP Codes. Based on data visualization and pairwise correlation analyses among ZIP Codes, we failed to find evidence in support of this hypothesis (Fig 5). In fact, influenza hospitalization patterns exhibited significantly more similarity within the lowest poverty quartile than within the less impoverished quartiles. To test for significance, we randomly assigned ZIP Codes to income quartiles 5,000 times and repeated the analysis. The observed mean correlation among the most impoverished quartiles was higher than all of the 5,000 randomizations (i.e., *p* < 0.0002) and the observed median was higher than all but 2 of the simulations (i.e., *p* = 0.0004).

For each poverty quartile, we also performed a principal-component analysis of ZIP Code level hospitalization counts. That is, we calculated the principal components of the matrix *Y*^(*i*)^ of hospitalization counts whose rows are weeks, and whose columns are the ZIP Codes within poverty quartile *i*. The highest poverty quartile has the highest percent variation explained by these leading components (33.2%), as compared to 13.2% in the least impoverished, 19.3% in the upper-middle, and 26.0% in the lower-middle quartiles, indicating greater synchrony in influenza trends within more impoverished populations. Thus, we do not believe that the reduced performance in lower socioeconomic groups stems from greater variation in temporal flu trends. We utilized the same permutation-based approach described above to test for a significant difference in the principle component analysis results (*p* < 0.001).

Next, we considered the hypothesis that geographic clustering might explain the discrepancies in forecast accuracy. One might intuitively expect nearby populations to exhibit similar influenza patterns; consequently, spatially aggregated populations should be more amenable to forecasting than more dispersed populations. We found that the lowest poverty ZIP Codes have similar patterns of spatial aggregation as the other quartiles (using both Moran’s I [36] and inter-centroid distances, see S5 Fig and S4 Text). However, the majority of these ZIP Codes are clustered in Dallas and Tarrant Counties, which is well represented in both our predictor and hospitalization data. To confirm that the uneven distribution of the poverty quartiles across counties (see S5 Fig) did not bias our results, we fit separate prediction models to Dallas and Tarrant counties. In both cases, we confirm our results that forecast accuracy decreases as poverty level increases (see S4 Text).

Finally, to further evaluate alternative explanations for the observed bias, we conducted a simulation experiment to address the role of other factors, such as reduced rates of ILI primary care in lower socioeconomic groups [37, 38], lower correlation between ILI-related Internet searches and actual ILI in lower socioeconomic groups [39, 40], socioeconomic differences in vaccination levels [41, 42], and/or socioeconomic differences in underlying health conditions [43]. The results of this simulation experiment demonstrate that, when all else is equal, a higher hospitalization rate should increase statistical power and provide greater prediction precision (S6 Text, S2 and S3 Figs).

## Discussion

Populations with lower socioeconomic status have higher hospitalization rates across a range of diseases [4, 44], caused in part by reduced access to healthcare [37]. Our analysis suggests a similar disparity in the accuracy of public health outbreak surveillance.

Specifically, a combination of clinical symptom reports, Internet searches, and electronic emergency room data can predict week-ahead inpatient influenza hospitalizations more reliably in higher socioeconomic than in lower socioeconomic populations. Given this performance discrepancy, we were surprised to find that high poverty ZIP Codes exhibit much more synchronous influenza hospitalization patterns than low poverty ZIP Codes and are geographically clustered. Thus, the failure likely stems from data bias or under-sampling of at-risk populations. We speculate that GFT (which tallies the number of influenza related Google searches) and ILINet (which collects data from volunteer outpatient clinics) provide low coverage of at-risk populations [5, 6, 45], while BioSense 2.0 may be biased by an excess in non-emergency visits to emergency rooms among uninsured and Medicaid recipients [46].

Over 100 years of epidemiological study demonstrates a consistent, positive association between health and economic prosperity [47, 48]. In many settings, lower socioeconomic status has been linked to both reduced access to healthcare and increased burden of both infectious and chronic diseases [37, 49–51]. For example, the REACH 2010 surveillance program in the U.S.A. found that, “More minorities reported being in fair or poor health, but they did not see a doctor because of the cost.” [49] and a recent study on neonatal intensive care in the US found that, “Black, Hispanic, and Asian infants were segregated across NICUs [neonatal intensive care units], reflecting the racial segregation of minority populations in the United States,” which translated into lower-quality care for infants in the most at-risk populations [52]. In this vein, we found a positive correlation between poverty and influenza hospitalization rates in study populations under age 65, which is consistent with a three-fold excess in pediatric influenza-related hospitalizations estimated for a Connecticut at-risk community [53]. However, it is unknown which of many possible factors—including differences in access to care, vaccine coverage, or prevalence of underlying conditions—are driving this disparity.

Our study identifies another related socioeconomic inequity—a reduced capability to detect and monitor outbreaks in at-risk populations—which impedes effective public health interventions. An analogous surveillance gap has been identified for cancer [54]. Ironically, surveillance systems seem to neglect communities most in need of intervention. New methods for designing and optimizing disease data collection have focused on state-level coverage [55–59] or assumed that risk was evenly spread across well-mixed populations [60], but could be adapted to identify data sources that remedy critical gaps or biases.

We recognize several important limitations of our study. First, our goal was to forecast inpatient hospitalizations for influenza. It is likely that different forms or amounts of bias might manifest themselves had we focused on a different objective. Second, our analysis was restricted to the Dallas-Fort Worth region from which we obtained BioSense 2.0 data, and may not generalize to the rest of the USA nor globally. Third, since we could not access BioSense 2.0 with influenza diagnoses, we used upper respiratory infection data as a proxy. We expect that influenza-specific BioSense 2.0 records would generally improve one-week-ahead predictions, but may or may not close the surveillance poverty gap. Fourth, we did not consider many other data sets, some of which might provide more representative coverage of at-risk populations, including public health laboratory data [61], pharmacy sales [62], school absenteeism records [63, 64], or other Internet-sourced or social media data [22, 65]. Fifth, because we used a lasso penalty to regularize the regression coefficients–implying that the number of degrees of freedom does not necessarily increase with the number of predictors–we could not apply standard model selection methods, such as Akaike Information Criteria, to compare the performance across models (rows of Table 2). Although BioSense 2.0 yields slightly higher performance scores across all poverty quartiles, we leave a definitive comparison among different combinations of surveillance data sources for future study. Sixth, we did not have individual-level patient socioeconomic and/or ZIP Code information from ILINet, BioSense 2.0, and GFT, and thus we were unable to assess directly whether lower socioeconomic groups are underrepresented. However, prior studies suggest that lower socioeconomic groups use the Internet less frequently than higher socioeconomic groups, and that disease-related signals derived from Internet-search data poorly reflect incidence in lower socioeconomic communities [39, 40, 45]. Interestingly, our results suggest that predictions based solely on GFT performed no worse in the lowest income quartile than did other candidate predictors. Researchers with access to individual-level BioSense 2.0 and GFT data–or other systems such as FilmArray Trend [66]–could test our hypothesis, and perhaps develop methods for subsampling the data to improve predictive performance in low income areas. Finally, the Texas inpatient hospitalization data did not indicate whether patients were admitted through an emergency department. Therefore, we were unable to determine whether visitation rate to emergency departments for influenza varied by socioeconomic status. We note that the majority of inpatient hospitalizations in the US are not preceded by an emergency department visit [67].

A growing community of researchers and practitioners across public health, medicine, science, military, and non-governmental organizations are developing and deploying technology-enabled surveillance systems [22] to support adaptive management of infectious diseases [68] and deliver actionable forecasts [69–76]. Many of these efforts focused on improving the timeliness and accuracy of bioevent detection, situational awareness, and forecasting [34, 77]. However, our results suggest a different, and arguably more important priority: improving coverage in at-risk populations. Gaps in both traditional and early next generation surveillance systems compound health disparities in populations with reduced access to healthcare or higher rates of severe disease. Thus, as surveillance systems are upgraded and expanded to incorporate novel data sources, and crowd-sourced/participatory systems are deployed, particular attention should be paid to improving equity, in addition to other performance goals [22, 25]. We further argue that our results highlight the critical need for more research into drivers of disease dynamics and studies to measure the burden of disease–across severity levels–in at-risk communities.

### Conclusions

We introduce a robust and flexible method for improving and bench marking situational awareness. Our method offers a general statistical model for short-term prediction, that can systematically integrate diverse data sources, including traditional surveillance data, electronic medical records and Internet-source digital data. We used the method to construct a surveillance system that made one-week-ahead predictions of influenza hospitalizations from real-time BioSense 2.0, Google Flu Trends and ILINet data. While overall performance was reasonable, we discovered a critical data vulnerability in Dallas-Fort Worth’s most at-risk populations. This surveillance design framework can be readily applied to evaluate and integrate new data sources that address this challenge.

## Supporting information

S1 Text This document contains supplemental information regarding the use of hospitalization forecasts as predictors. (PDF)

S2 Text This document contains supplemental information detailing additional statistical model fits and evaluations. (PDF)

S3 Text This document contains supplemental information regarding subsampling residuals to test for model bias. (PDF)

S4 Text This document contains supplemental information regarding models fit only to Dallas and Tarrant County hospitalizations and a spatial autocorrelation analysis using Moran’s I. (PDF)

S5 Text This document contains supplemental information regarding models fit using ZIP Code level ILINet data. (PDF)

S6 Text This document contains supplemental information regarding simulations to evaluate the sensitivity of model predictions to hospitalization rate and surveillance detection rate. (PDF)

S1 Fig Result of the permutation test for the ILI + BioSense + GFT model across 10,000 Monte Carlo samples. The vertical red line is at 3.3, the observed value based on the poverty grouping. The results indicate that it is unlikely for the observed value to arise by chance. The Monte Carlo p-value is 0.0001, with only of our randomized permutations yielding an ORMSE gap at least as large as 3.3.
(TIF)

S2 Fig Simulating disparate case hospitalization rates. The curves illustrate a typical simulation. The left-hand panel depicts the Influenza-Like-Illness (ILI) time series (blue) for populations A and B, and surveillance time series for A (red) and B (green) derived by stochastically sampling the ILI time series, assuming that 10% of cases are detected by the system (for example, via internet use or physician visits). The right-hand panel depicts the hospitalization time series and predicted hospitalizations for populations A and B, which had hospitalization rates of 0.1 and 0.9, respectively. The hospitalization curves were generated by stochastically sampling the ILI curve in the left-hand panel and the predictions were created using the same regression model as in the main analysis. The average *R*^2^ over 10,000 simulations for these predictions are 0.9986 and 0.9993, for A and B, respectively.
(TIF)

S3 Fig As the hospitalization rate or the surveillance detection rate drops, the predictions become less precise. For each combination of surveillance detection rate and hospitalization rate, we run 100 simulations to estimate the expected *R*^2^. These simulations are conducted assuming *β* = 0.076 and *γ* = 0.07 and the results are qualitatively the same for other values of these parameters.
(TIF)

S4 Fig Comparison between one-week ahead model predictions and the total number of weekly observed influenza hospitalizations for each of the four poverty quartiles (A) upper quartile, (B) upper-middle quartile, (C) lower-middle quartile, (D) lowest quartile and the distribution of out-of-sample prediction errors (observed—predicted) for the (E) upper quartile, (F) upper-middle quartile, (G) lower-middle quartile, and (H) lowest quartile. Across all four quartiles, the model was unbiased according to a resampling test on the residuals.
(TIF)

S5 Fig Geographic distribution of ZIP Codes by poverty quartile. A. Boxplots of pairwise distances between ZIP Codes in the four poverty quartiles. ZIP Codes in the highest poverty quartile (red) are significantly closer than ZIP Codes in the other three quartiles (ANOVA and Tukey Honest Test *p* < 0.001). B. Distribution of ZIP Codes in each poverty quartile by county. The most impoverished quartile (21-48%) is over-represented in Dallas County.
(TIF)
