# Supplementary material for: Socioeconomic bias in influenza surveillance
Source: PLoS Comput Biol. 2020 Jul 9;16(7):e1007941. doi: 10.1371/journal.pcbi.1007941 (PMC7347107; doi:10.1371/journal.pcbi.1007941)
Supplement: S1 Text — (PDF) [file pcbi.1007941.s001.pdf]

Supplemental Text 1:
Socioeconomic bias in influenza surveillance

Samuel V. Scarpino[1,2], James G. Scott[3], Rosalind M. Eggo[4], Bruce Clements[5], Nedialko B. Dimitrov[3], and Lauren Ancel Meyers[3,6,*]

[1]Northeastern University, Boston, MA, 02115, USA
[2]ISI Foundation, 10126 Turin, Italy
[3]The University of Texas at Austin, Austin, TX, USA
[4]London School of Hygiene and Tropical Medicine, London, UK
[5]Pediatric Healthcare Connection, Austin, TX, USA
[6]Santa Fe Institute, Santa Fe, New Mexico, USA
[*]address general correspondence to laurenmeyers@austin.utexas.edu

Including hospitalization forecasts as predictors

One additional set of predictors we could have made available to the forecasting models were the out-of-sample hospitalization forecasts. Given the high-degree of accuracy in the three lower poverty quartiles, it is possible that these forecasts could serve as useful predictors in the highest poverty quartile. To evaluate whether including hospitalization forecasts might partially alleviate the bias, we ran the following experiment: 1.) Following the methodology in our paper, we derive best-fit, out-of-sample forecasts for hospitalizations in each of the three lower poverty quartiles; 2.) we fit the predictive model for the lowest income quartile, allowing the model to select predictors that include all of the original variables and the influenza hospitalization forecasts in the other quartiles; 3.) we compare the out-of-sample accuracy for this expanded model to predictions made using the original model, i.e., a model without the hospitalization forecasts included as possible predictors. What we find is that the out-of-sample estimates were worse in this expanded model, as compared to the original model (See Table 1). This result suggests that including hospitalization forecasts from other poverty quartiles cannot alleviate the bias present in the highest poverty quartile.

	Without Nowcast Hospitalizations	With Nowcast Hospitalizations
ILI	3.19	4.46
BioSense	3.40	6.54
GFT	2.91	6.81
ILI + BioSense	3.13	6.36
ILI + GFT	2.76	3.33
BioSense + GFT	3.21	3.95
ILI + BioSense + GFT	3.21	4.65

Table 1. Table presents the out-of-sample (leave-one-out) root mean-squared error (ORMSE) for the highest poverty quartile resulting from two Poisson generalized additive models. In the first model (Without Nowcast Hospitalizations), only the original variables were available for the model; however, in the second model (With Nowcast Hospitalizations) we include the hospitalization forecasts for the three lower poverty quartiles. The model that *excluded* the hospitalization forecasts, i.e., the model presented in the main text of the manuscript, outperformed the model that included hospitalization forecasts. Values are normalized by the population size of the quartile and were multiplied by 10^6 to obtain an ORMSE per one million residents.
