# Supplementary material for: Socioeconomic bias in influenza surveillance
Source: PLoS Comput Biol. 2020 Jul 9;16(7):e1007941. doi: 10.1371/journal.pcbi.1007941 (PMC7347107; doi:10.1371/journal.pcbi.1007941)
Supplement: S2 Text — (PDF) [file pcbi.1007941.s002.pdf]

Supplemental Text 2:
Socioeconomic bias in influenza surveillance

Samuel V. Scarpino[1,2], James G. Scott[3], Rosalind M. Eggo[4], Bruce Clements[5], Nedialko B. Dimitrov[3], and Lauren Ancel Meyers[3,6,*]

[1]Northeastern University, Boston, MA, 02115, USA
[2]ISI Foundation, 10126 Turin, Italy
[3]The University of Texas at Austin, Austin, TX, USA
[4]London School of Hygiene and Tropical Medicine, London, UK
[5]Pediatric Healthcare Connection, Austin, TX, USA
[6]Santa Fe Institute, Santa Fe, New Mexico, USA
[*]address general correspondence to laurenmeyers@austin.utexas.edu

Additional statistical model fits and evaluations

In addition, we fit Poisson, negative binomial, and Gaussian models using one-week-ahead and two-week-ahead forecasts and evaluated each model using leave-one-out root-mean-square-error and log-likelihood and 60/40 training testing root-mean-square-error and log-likelihood. The lowest income quartile performed significantly worse across all models considered.

Generalized additive models

	Poverty Quartile				
Surveillance Data Sources	1st quartile (lowest poverty)	2nd quartile	3rd quartile	4th quartile (highest poverty)	Aggregate
ILI	-243.20	-268.55	-323.38	-449.58	-191.60
BioSense	-252.47	-278.49	-326.32	-451.25	-211.57
GFT	-227.45	-241.87	-311.60	-375.69	-160.17
ILI + BioSense	-243.19	-261.42	-301.16	-416.81	-173.76
ILI + GFT	-223.72	-240.27	-322.68	-354.08	-154.03
BioSense + GFT	-231.61	-257.83	-317.07	-415.65	-182.16
ILI + BioSense + GFT	-231.61	-257.83	-297.48	-415.65	-182.16

Table 1. The table shows out-of-sample (leave-one-out) Poisson log-likelihood using a Poisson generalized additive model. The values were normalized by the population size of each quantile and then multiplied by 10^6, so are Poisson log-likelihood per one million residents. The rightmost column gives aggregate ORMSE across all ZIP Codes included in our study area. The quartiles contained: [0-8) (1st quartile), [8-12) (2nd quartile), [12-21) (3rd quartile), and > 21 (4th quartile) percent of residents below the poverty line.

Surveillance Data Sources	Poverty Quartile<br>1st quartile (lowest poverty)	2nd quartile	3rd quartile	4th quartile (highest poverty)	Aggregate
ILI	1.47	1.80	2.60	3.93	2.17
BioSense	1.64	1.91	2.60	3.68	2.28
GFT	1.24	1.62	2.50	3.45	1.85
ILI + BioSense	1.51	1.81	2.46	3.58	2.04
ILI + GFT	1.19	1.56	2.56	3.27	1.91
BioSense + GFT	1.34	1.70	2.50	3.55	2.04
ILI + BioSense + GFT	1.34	1.70	2.39	3.55	2.04

Table 2. The table shows out-of-sample (leave-one-out) root mean-squared error (RMSE) using a Gaussian generalized additive model.The values were normalized by the population size of each quantile and then multiplied by 10^6, so are the ORMSE per one million residents. The rightmost column gives aggregate ORMSE across all ZIP Codes included in our study area. The quartiles contained: [0-8) (1st quartile), [8-12) (2nd quartile), [12-21) (3rd quartile), and $>$ 21 (4th quartile) percent of residents below the poverty line.

Surveillance Data Sources	Poverty Quartile<br>1st quartile (lowest poverty)	2nd quartile	3rd quartile	4th quartile (highest poverty)	Aggregate
ILI	-250.01	-273.16	-322.71	-447.12	-192.58
BioSense	-253.13	-278.45	-346.70	-480.60	-199.57
GFT	-231.79	-240.43	-311.57	-382.54	-159.35
ILI + BioSense	-255.92	-271.01	-324.76	-438.27	-177.71
ILI + GFT	-226.35	-250.27	-326.13	-405.46	-176.10
BioSense + GFT	-228.51	-251.79	-324.07	-393.63	-165.57
ILI + BioSense + GFT	-228.51	-251.79	-303.11	-393.63	-165.57

Table 3. The table shows out-of-sample (leave-one-out) Poisson log-likelihood using a Gaussian generalized additive model. The values were normalized by the population size of each quantile and then multiplied by 10^6, so are Poisson log-likelihood per one million residents. The rightmost column gives aggregate ORMSE across all ZIP Codes included in our study area. The quartiles contained: [0-8) (1st quartile), [8-12) (2nd quartile), [12-21) (3rd quartile), and $>$ 21 (4th quartile) percent of residents below the poverty line.

Generalized linear models

Surveillance Data Sources	Poverty Quartile<br>1st quartile (lowest poverty)	2nd quartile	3rd quartile	4th quartile (highest poverty)	Aggregate
ILI	1.74	2.16	3.01	4.35	2.56
Biosense	2.32	2.87	3.34	3.84	2.91
GFT	1.81	2.02	2.89	3.78	2.51
ILI + Biosense	1.93	2.87	3.18	3.92	3.09
ILI + GFT	1.83	2.26	3.30	4.27	2.76
Biosense + GFT	1.49	2.01	3.78	4.01	2.86
ILI + Biosense + GFT	1.49	2.01	3.51	4.01	2.86

Table 4. Out-of-sample (leave-one-out) root mean-squared error (ORMSE) for each Poisson generalized linear model. Values are normalized by the population size of each ZIP Code quartile and then multiplied by 10^6 to obtain ORMSE per one million residents. The rightmost column gives aggregate ORMSE across all ZIP Codes included in our study area. The quartiles contained: [0-8) (1st quartile), [8-12) (2nd quartile), [12-21) (3rd quartile), and > 21 (4th quartile) percent of residents below the poverty line.

Surveillance Data Sources	Poverty Quartile<br>1st quartile (lowest poverty)	2nd quartile	3rd quartile	4th quartile (highest poverty)	Aggregate
ILI	-283.81	-331.86	-387.64	-530.89	-256.24
Biosense	-279.47	-297.42	-336.19	-454.66	-217.10
GFT	-241.61	-283.14	-316.04	-401.24	-183.36
ILI + Biosense	-275.70	-296.73	-330.79	-455.31	-214.60
ILI + GFT	-244.30	-279.42	-329.29	-417.81	-191.45
Biosense + GFT	-249.45	-283.79	-329.83	-435.15	-202.94
ILI + Biosense + GFT	-249.45	-283.79	-334.04	-435.45	-202.94

Table 5. The table shows out-of-sample (leave-one-out) Poisson log-likelihood using a Poisson generalized linear model. The values were normalized by the population size of each quantile and then multiplied by 10^6, so are Poisson log-likelihood per one million residents. The rightmost column gives aggregate ORMSE across all ZIP Codes included in our study area. The quartiles contained: [0-8) (1st quartile), [8-12) (2nd quartile), [12-21) (3rd quartile), and > 21 (4th quartile) percent of residents below the poverty line.

Surveillance Data Sources	Poverty Quartile<br>1st quartile (lowest poverty)	2nd quartile	3rd quartile	4th quartile (highest poverty)	Aggregate
ILI	7.57	12.87	24.01	32.11	25.39
Biosense	3.22	5.47	5.97	26.24	13.37
GFT	2.70	7.41	7.85	12.90	8.72
ILI + Biosense	8.45	7.20	11.90	32.44	21.83
ILI + GFT	3.79	5.36	7.46	14.41	8.51
Biosense + GFT	3.41	9.36	7.69	25.23	15.60
ILI + Biosense + GFT	3.41	9.36	8.13	25.23	15.60

Table 6. The table shows out-of-sample (leave-one-out) root mean-squared error (RMSE) using a negative binomial generalized linear model.The values were normalized by the population size of each quantile and then multiplied by 10^6, so are the ORMSE per one million residents. The rightmost column gives aggregate ORMSE across all ZIP Codes included in our study area. The quartiles contained: [0-8) (1st quartile), [8-12) (2nd quartile), [12-21) (3rd quartile), and > 21 (4th quartile) percent of residents below the poverty line.

Surveillance Data Sources	Poverty Quartile<br>1st quartile (lowest poverty)	2nd quartile	3rd quartile	4th quartile (highest poverty)	Aggregate
ILI	-402.34	-574.01	-816.12	-1148.87	-749.84
Biosense	-290.94	-335.62	-383.02	-801.13	-381.62
GFT	-253.32	-377.78	-421.32	-590.50	-312.02
ILI + Biosense	-359.66	-375.78	-509.79	-933.10	-550.78
ILI + GFT	-269.68	-355.46	-427.58	-642.05	-329.31
Biosense + GFT	-269.37	-395.87	-399.05	-822.63	-427.77
ILI + Biosense + GFT	-269.37	-395.87	-410.92	-822.63	-427.77

Table 7. The table shows out-of-sample (leave-one-out) Poisson log-likelihood using a negative binomial generalized linear model. The values were normalized by the population size of each quantile and then multiplied by 10^6, so are Poisson log-likelihood per one million residents. The rightmost column gives aggregate ORMSE across all ZIP Codes included in our study area. The quartiles contained: [0-8) (1st quartile), [8-12) (2nd quartile), [12-21) (3rd quartile), and > 21 (4th quartile) percent of residents below the poverty line.
