# Supplementary material for: Socioeconomic bias in influenza surveillance
Source: PLoS Comput Biol. 2020 Jul 9;16(7):e1007941. doi: 10.1371/journal.pcbi.1007941 (PMC7347107; doi:10.1371/journal.pcbi.1007941)
Supplement: S3 Text — (PDF) [file pcbi.1007941.s003.pdf]

Supplemental Text 3:
Socioeconomic bias in influenza surveillance

Samuel V. Scarpino[1,2], James G. Scott[3], Rosalind M. Eggo[4], Bruce Clements[5], Nedialko B. Dimitrov[3], and Lauren Ancel Meyers[3,6,*]

[1]Northeastern University, Boston, MA, 02115, USA
[2]ISI Foundation, 10126 Turin, Italy
[3]The University of Texas at Austin, Austin, TX, USA
[4]London School of Hygiene and Tropical Medicine, London, UK
[5]Pediatric Healthcare Connection, Austin, TX, USA
[6]Santa Fe Institute, Santa Fe, New Mexico, USA
[*]address general correspondence to laurenmeyers@austin.utexas.edu

Subsampling residuals

	Mean	Upper 95th percentile	Lower 95th percentile
1st quartile	0.49	0.60	0.38
2nd quartile	0.50	0.61	0.39
3rd quartile	0.47	0.60	0.36
4the quartile	0.48	0.62	0.36

Table 1. The mean and 95% confidence intervals for the proportion of the residual error that was positive (over prediction) determined by bootstrapping the residuals 1,000 times for the best-fit model in each quartile. Across all four quartiles, with the 1st having the lowest proportion below poverty and 4th the highest, the best-fit models were unbiased. A value of 0.5 would indicate that exactly half of the error was associated with under-prediction and half of the error was associated with over-prediction.
