# Supplementary material for: Socioeconomic bias in influenza surveillance
Source: PLoS Comput Biol. 2020 Jul 9;16(7):e1007941. doi: 10.1371/journal.pcbi.1007941 (PMC7347107; doi:10.1371/journal.pcbi.1007941)
Supplement: S4 Text — (PDF) [file pcbi.1007941.s004.pdf]

Supplemental Text 4:
Socioeconomic bias in influenza surveillance

Samuel V. Scarpino[1,2], James G. Scott[3], Rosalind M. Eggo[4], Bruce Clements[5], Nedialko B. Dimitrov[3], and Lauren Ancel Meyers[3,6,*]

[1]Northeastern University, Boston, MA, 02115, USA
[2]ISI Foundation, 10126 Turin, Italy
[3]The University of Texas at Austin, Austin, TX, USA
[4]London School of Hygiene and Tropical Medicine, London, UK
[5]Pediatric Healthcare Connection, Austin, TX, USA
[6]Santa Fe Institute, Santa Fe, New Mexico, USA
[*]address general correspondence to laurenmeyers@austin.utexas.edu

Fitting only to Dallas and Tarrant Counties and spatial auto-correlation analysis

	Poverty Quartile				
Surveillance Data Sources	1st quartile (lowest poverty)	2nd quartile	3rd quartile	4th quartile (highest poverty)	Aggregate
ILI	0.61	1.20	1.73	3.19	1.48
BioSense	0.62	1.27	1.62	3.40	1.65
GFT	0.59	1.16	1.76	2.91	1.29
ILI + BioSense	0.62	1.19	1.62	3.13	1.50
ILI + GFT	0.58	1.16	1.62	2.76	1.19
BioSense + GFT	0.60	1.17	1.75	3.21	1.55
ILI + BioSense + GFT	0.60	1.17	1.75	3.21	1.55

Table 1. Out-of-sample (leave-one-out) root mean-squared error (ORMSE) for each Poisson generalized additive model fit only ZIP Codes in Dallas county. Values are normalized by the population size of each ZIP Code quartile and then multiplied by 10^6 to obtain ORMSE per one million residents. The rightmost column gives aggregate ORMSE across all Dallas county ZIP Codes. The quartiles contained: [0-8) (1st quartile), [8-12) (2nd quartile), [12-21) (3rd quartile), and > 21 (4th quartile) percent of residents below the poverty line.

Surveillance Data Sources	Poverty Quartile<br>1st quartile (lowest poverty)	2nd quartile	3rd quartile	4th quartile (highest poverty)	Aggregate
ILI	0.72	0.69	0.85	1.00	0.57
BioSense	0.79	0.72	0.81	1.04	0.54
GFT	0.65	0.57	0.84	0.94	0.54
ILI + BioSense	0.66	0.70	0.81	0.99	0.58
ILI + GFT	0.77	0.54	0.80	0.98	0.56
BioSense + GFT	0.62	0.70	0.81	0.99	0.59
ILI + BioSense + GFT	0.69	0.70	0.81	0.99	0.52

Table 2. Out-of-sample (leave-one-out) root mean-squared error (ORMSE) for each Poisson generalized additive model fit only ZIP Codes in Tarrant county. Values are normalized by the population size of each ZIP Code quartile and then multiplied by 10^6 to obtain ORMSE per one million residents. The rightmost column gives aggregate ORMSE across all Tarrant county ZIP Codes. The quartiles contained: [0-8) (1st quartile), [8-12) (2nd quartile), [12-21) (3rd quartile), and > 21 (4th quartile) percent of residents below the poverty line.

Quartile(s)	Moran's I (nearest)	p value* (nearest)	Moran's I (10Km)	p value* (10Km)
A	0.09	1.00	0.05	1.00
B	0.29	0.12	0.18	0.30
C	0.07	1.00	0.14	0.80
D	0.01	1.00	0.03	1.00
A+B	0.16	0.27	0.16	0.05
C+D	0.16	0.21	0.09	0.38
A+B+C	0.29	0.01	0.24	0.01
A+C+D	0.54	0.01	0.42	0.01
A+B+D	0.60	0.01	0.50	0.01
B+C+D	0.36	0.01	0.26	0.01
A+B+C+D	0.50	0.01	0.39	0.01

Table 3. To determine whether differences in spatial autocorrelation of income may have influenced forecast accuracy, we calculated Moran's I for all combinations of income quartile and using both nearest neighbor and continuous weighting based on each ZIP Code's centroid. Briefly, we find evidence that spatial autocorrelation in income exists but does not seem to be stronger in any one particular quartile. Calculations were performed in the R package spdep using 1,000 Monte Carlo simulations to determine statistical significance. We also explored larger and smaller windows for the continuous distance averaging and find similar support for distances up to 50kM. *p values were adjusted for multiple comparisons using a Bonferroni correction.
