# Supplementary material for: Socioeconomic bias in influenza surveillance
Source: PLoS Comput Biol. 2020 Jul 9;16(7):e1007941. doi: 10.1371/journal.pcbi.1007941 (PMC7347107; doi:10.1371/journal.pcbi.1007941)
Supplement: S5 Text — (PDF) [file pcbi.1007941.s005.pdf]

Supplemental Text 5: Socioeconomic bias in influenza surveillance

Samuel V. Scarpino[1,2], James G. Scott[3], Rosalind M. Eggo[4], Bruce Clements[5], Nedialko B. Dimitrov[3], and Lauren Ancel Meyers[3,6,*]

[1]Northeastern University, Boston, MA, 02115, USA
[2]ISI Foundation, 10126 Turin, Italy
[3]The University of Texas at Austin, Austin, TX, USA
[4]London School of Hygiene and Tropical Medicine, London, UK
[5]Pediatric Healthcare Connection, Austin, TX, USA
[6]Santa Fe Institute, Santa Fe, New Mexico, USA
[*]address general correspondence to laurenmeyers@austin.utexas.edu

Models fit using ZIP Code level ILINet data.

Surveillance Data Sources	Poverty Quartile: 1st quartile (lowest poverty)	2nd quartile	3rd quartile	4th quartile (highest poverty)	Aggregate
ILI	1.63	1.87	2.80	3.14	2.11
Biosense	1.63	2.12	2.83	3.99	2.52
GFT	1.28	1.70	2.78	3.43	2.00
ILI + Biosense	1.72	1.77	2.42	3.41	1.94
ILI + GFT	1.47	1.71	2.42	2.59	1.66
Biosense + GFT	1.34	1.97	2.75	3.80	2.35
ILI + Biosense + GFT	1.34	1.70	2.27	3.31	1.56

Table 1. Out-of-sample (leave-one-out) root mean-squared error (ORMSE) for each Poisson generalized additive model with ZIP Code level data from ILINet as a possible predictor. Values are normalized by the population size of each ZIP Code quartile and then multiplied by 10^6 to obtain ORMSE per one million residents. The rightmost column gives aggregate ORMSE across all ZIP Codes in the six county area. The quartiles contained: [0-8) (1st quartile), [8-12) (2nd quartile), [12-21) (3rd quartile), and >21 (4th quartile) percent of residents below the poverty line.
