# Supplementary material for: Socioeconomic bias in influenza surveillance
Source: PLoS Comput Biol. 2020 Jul 9;16(7):e1007941. doi: 10.1371/journal.pcbi.1007941 (PMC7347107; doi:10.1371/journal.pcbi.1007941)
Supplement: S6 Text — (PDF) [file pcbi.1007941.s006.pdf]

Supplemental Text 6: Socioeconomic bias in influenza surveillance

Samuel V. Scarpino[1,2], James G. Scott[3], Rosalind M. Eggo[4], Bruce Clements[5], Nedialko B. Dimitrov[3], and Lauren Ancel Meyers[3,6,*]

[1]Northeastern University, Boston, MA, 02115, USA
[2]ISI Foundation, 10126 Turin, Italy
[3]The University of Texas at Austin, Austin, TX, USA
[4]London School of Hygiene and Tropical Medicine, London, UK
[5]Pediatric Healthcare Connection, Austin, TX, USA
[6]Santa Fe Institute, Santa Fe, New Mexico, USA
[*]address general correspondence to laurenmeyers@austin.utexas.edu

Sensitivity of model predictions to hospitalization rate and surveillance detection rate.

Lower income groups often have a higher hospitalization rates than higher income groups (as illustrated in our Figure 3 and discussed in [1–3]). However, a higher case hospitalization rate, on its own, cannot explain the observed disparity in predictability of the time series across different poverty quartiles. Therefore, other factors must be at work, such as reduced rates of ILI primary care in lower socioeconomic groups [4, 5], lower correlation between ILI-related internet searches and actual ILI in lower socioeconomic groups [6], socioeconomic differences in vaccination levels [7, 8], or socioeconomic differences in underlying health conditions [9].

To demonstrate that a higher case hospitalization rate alone cannot explain the observed disparity in predictions between high poverty and low poverty quartiles, we conduct a simple simulation experiment. The simulation demonstrates that, when all else is equal, a higher hospitalization rate should instead lead to greater prediction accuracy. The larger number of hospitalizations increases the statistical power of the models. We now describe this analysis.

We used the following numerical simulation to assess whether a higher hospitalization rate in lower income populations might explain the reduction in hospital prediction accuracy (as opposed to biased sampling in surveillance data sets):

1. We generated a time series of Influenza-Like-Illness (ILI) cases reported per day, S. This time series was based on a standard Susceptible-Infectious-Recovered, i.e. SIR, model with a population of 100,000 individuals, where the force of infection, β, was 0.5, and the recovery rate, γ, was 0.4. The choice of these parameter values does not impact our qualitative results (Supplement Figure 2).

2. We create two populations, A and B, each with ILI trends that perfectly match S.

3. We simulate two *noisy* surveillance time series, one for population A and one for population B. For each day in each time series, we generate a binomial random variable,

with parameters n (number of trials) equal to the total number of ILI cases reported on that day and binomial probability p equal to 0.1. This assumes that the surveillance systems in both populations pick up cases stochastically at the same rate (10%). We refer to this parameter as the surveillance detection rate.

4. We simulate two hospitalization time series, one for population A and one for population B. As in step 3, for each day in each time series, we generate a binomial random variable with n (number of trials) equal to the total number of ILI cases reported on that day. In this case, however, the binomial probability models the hospitalization rate rather than the surveillance detection rate, and we assume that it differs between the two populations. Specifically, we use $p = 0.1$ and $p = 0.9$ in populations A and B, respectively.

5. We then attempt to predict the hospitalizations in both groups using the same regression model as in the main analysis.

We performed 10,000 stochastic simulations and found that predictions were better, on average, for the population with the higher case hospitalization rate than the population with the lower case hospitalization rate. Specifically, when comparing the predicted hospitalization time series to the actual hospitalization time series, the average R^2 was 0.9830 and 0.9914, for populations A and B, respectively – a statistically significant difference with $p < 0.0001$.

We modified the simulation described above to assess, more generally, the combined impact of the hospitalization rate and the surveillance detection rate on the prediction error. Specifically, we ran a total of 10,000 single population simulations, varying the case hospitalization rate and the surveillance detection rate from 0.1 to 0.9, assuming $\beta = 0.076$ and $\gamma = 0.07$. We find a marked and predictable decline in precision, as the hospitalization rate and surveillance detection rate decline (Supplement Figure 4), suggesting low sampling by a surveillance system (for example, stemming from reduced use of primary care and internet resources in disadvantaged populations), with or without an increase in hospitalization rate, could impede situational awareness and short-term forecasts.

References

[1] Thompson WW, Shay DK, Weintraub E, Brammer L, Bridges CB, Cox NJ, et al. Influenza-associated hospitalizations in the United States. Jama. 2004;292(11):1333–1340.

[2] Sloan C, Chandrasekhar R, Mitchel E, Schaffner W, Lindegren ML. Socioeconomic disparities and influenza hospitalizations, Tennessee, USA. Emerging infectious diseases. 2015;21(9):1602.

[3] Tam K, Yousey-Hindes K, Hadler JL. Influenza-related hospitalization of adults associated with low census tract socioeconomic status and female sex in New Haven County, Connecticut, 2007-2011. Influenza and other respiratory viruses. 2014;8(3):274–281.

[4] Shi L, Starfield B, Kennedy B, Kawachi I. Income inequality, primary care, and health indicators.(Original Research). Journal of Family Practice. 1999;48(4):275–285.

[5] Wilkinson RG, Pickett KE. Income inequality and population health: a review and explanation of the evidence. Social science & medicine. 2006;62(7):1768–1784.

[6] Richiardi L, Pizzi C, Paolotti D. Internet-based Epidemiology. In: Handbook of Epidemiology. Springer; 2014. p. 439–469.

[7] Fiscella K, Franks P, Gold MR, Clancy CM. Inequality in quality: addressing socioeconomic, racial, and ethnic disparities in health care. Jama. 2000;283(19):2579–2584.

[8] Galea S, Ahern J, Karpati A. A model of underlying socioeconomic vulnerability in human populations: evidence from variability in population health and implications for public health. Social science & medicine. 2005;60(11): 2417–2430.

[9] Adler NE, Newman K. Socioeconomic disparities in health: pathways and policies. Health affairs. 2002;21(2):60–76.
