# Supplementary figures and images for: Socioeconomic bias in influenza surveillance
Source: PLoS Comput Biol. 2020 Jul 9;16(7):e1007941. doi: 10.1371/journal.pcbi.1007941 (PMC7347107; doi:10.1371/journal.pcbi.1007941)

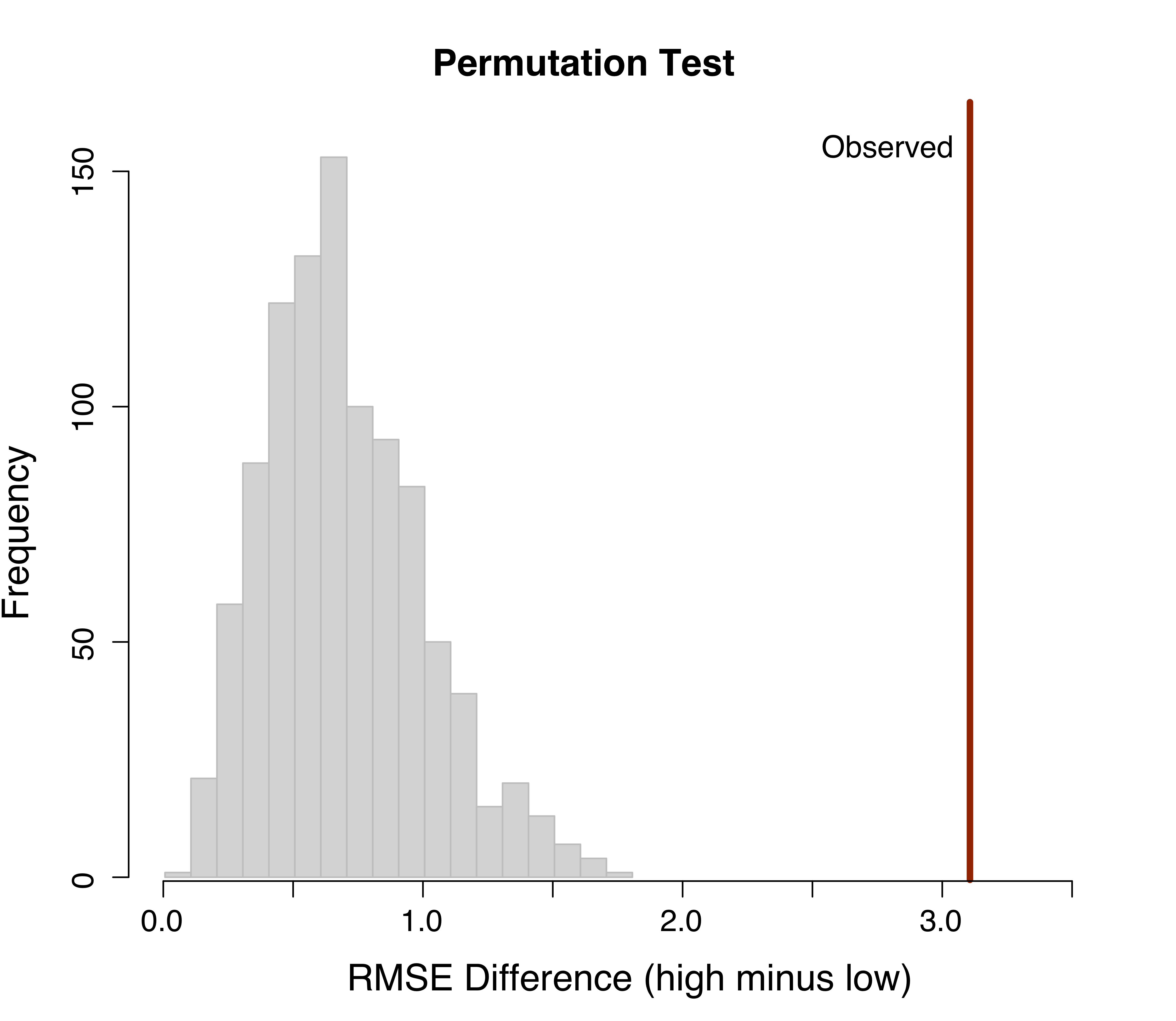

Supplement: S1 Fig — The vertical red line is at 3.3, the observed value based on the poverty grouping. The results indicate that it is unlikely for the observed value to arise by chance. The Monte Carlo p-value is 0.0001, with only of our randomized permutations yielding an ORMSE gap at least as large as 3.3. (TIF) [file pcbi.1007941.s007.tif]

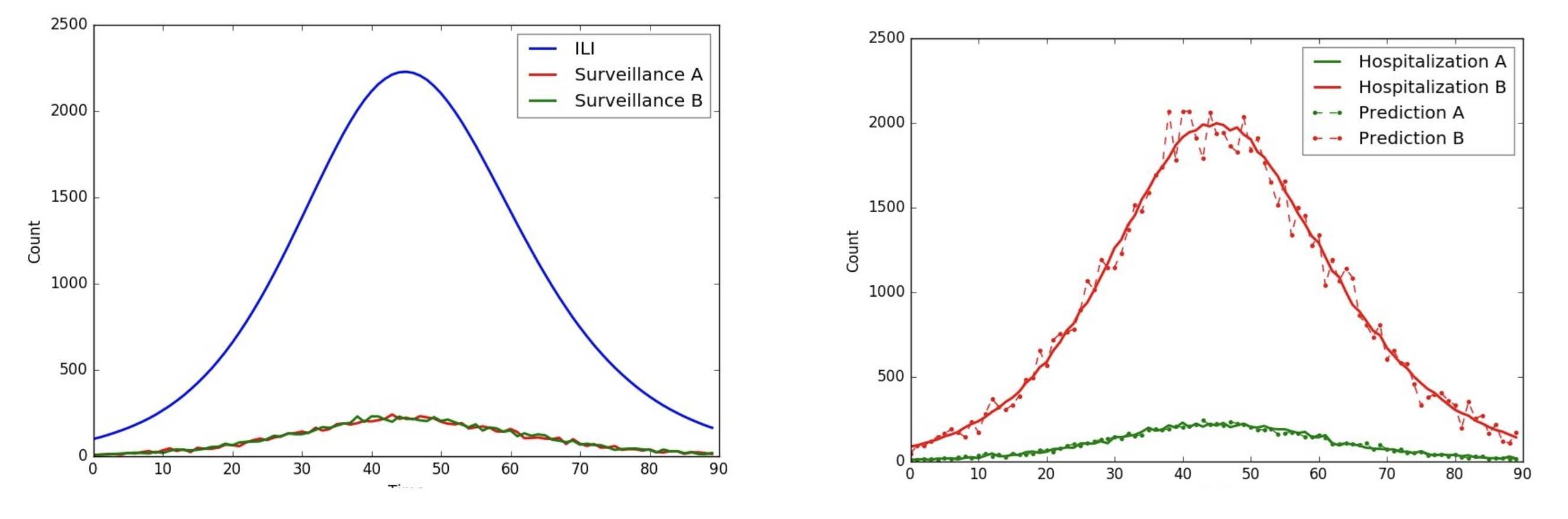

Supplement: S2 Fig — The curves illustrate a typical simulation. The left-hand panel depicts the Influenza-Like-Illness (ILI) time series (blue) for populations A and B, and surveillance time series for A (red) and B (green) derived by stochastically sampling the ILI time series, assuming that 10% of cases are detected by the system (for example, via internet use or physician visits). The right-hand panel depicts the hospitalization time series and predicted hospitalizations for populations A and B, which had hospitalization rates of 0.1 and 0.9, respectively. The hospitalization curves were generated by stochastically sampling the ILI curve in the left-hand panel and the predictions were created using the same regression model as in the main analysis. The average R2 over 10,000 simulations for these predictions are 0.9986 and 0.9993, for A and B, respectively. (TIF) [file pcbi.1007941.s008.tif]

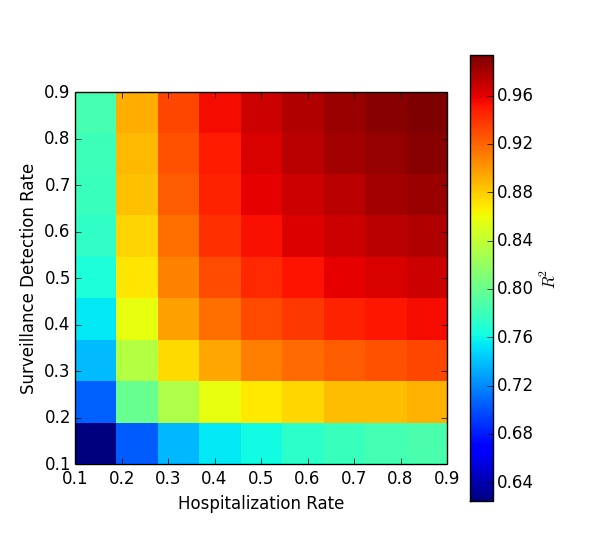

Supplement: S3 Fig — For each combination of surveillance detection rate and hospitalization rate, we run 100 simulations to estimate the expected R2. These simulations are conducted assuming β = 0.076 and γ = 0.07 and the results are qualitatively the same for other values of these parameters. (TIF) [file pcbi.1007941.s009.tif]

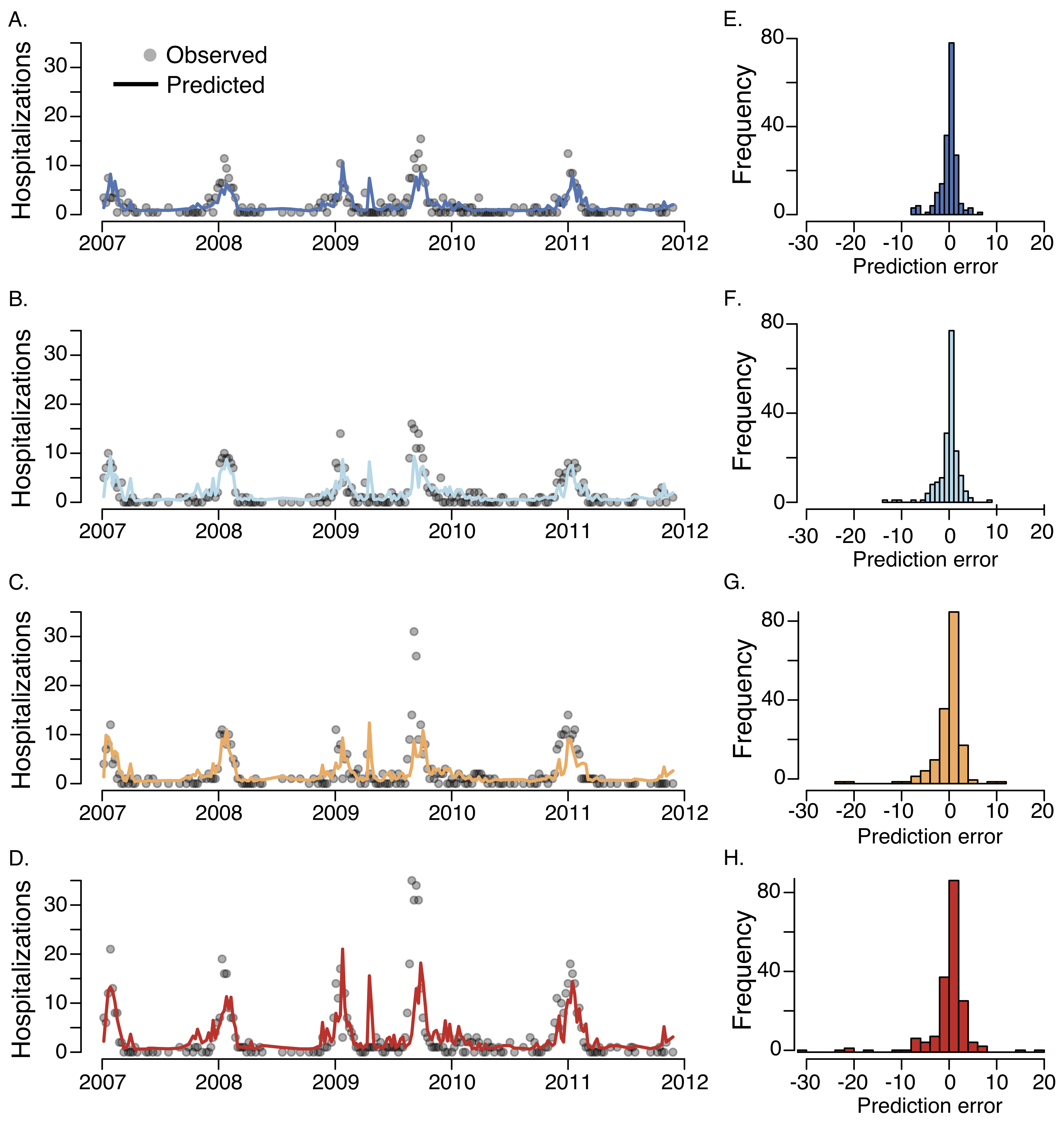

Supplement: S4 Fig — Across all four quartiles, the model was unbiased according to a resampling test on the residuals. (TIF) [file pcbi.1007941.s010.tif]

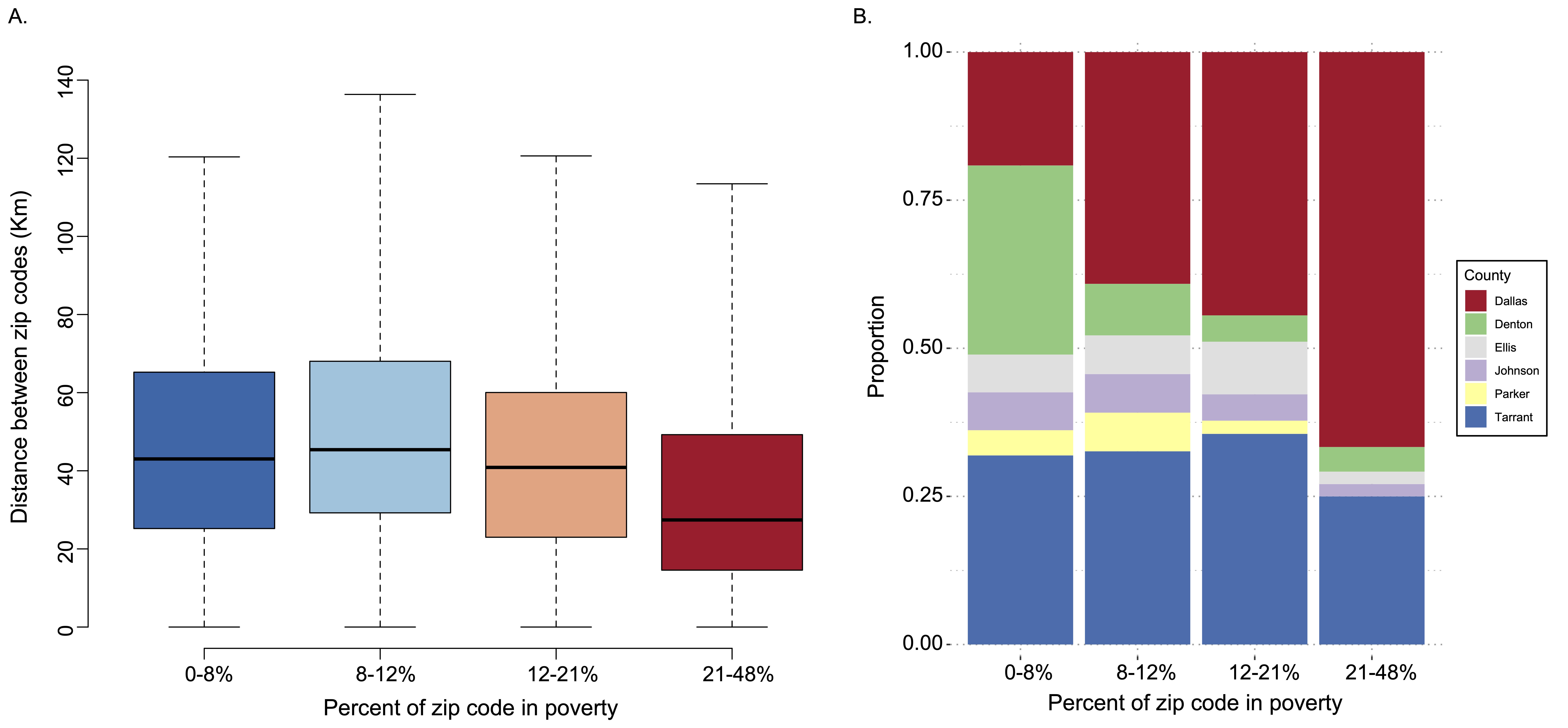

Supplement: S5 Fig — A. Boxplots of pairwise distances between ZIP Codes in the four poverty quartiles. ZIP Codes in the highest poverty quartile (red) are significantly closer than ZIP Codes in the other three quartiles (ANOVA and Tukey Honest Test p < 0.001). B. Distribution of ZIP Codes in each poverty quartile by county. The most impoverished quartile (21-48%) is over-represented in Dallas County. (TIF) [file pcbi.1007941.s011.tif]
